# Identification of Candidate Genes for Reactivity in Guzerat (*Bos indicus*) Cattle: A Genome-Wide Association Study

**DOI:** 10.1371/journal.pone.0169163

**Published:** 2017-01-26

**Authors:** Fernanda Caroline dos Santos, Maria Gabriela Campolina Diniz Peixoto, Pablo Augusto de Souza Fonseca, Maria de Fátima Ávila Pires, Ricardo Vieira Ventura, Izinara da Cruz. Rosse, Frank Angelo Tomita Bruneli, Marco Antonio Machado, Maria Raquel Santos Carvalho

**Affiliations:** 1 Departamento de Biologia Geral, Universidade Federal de Minas Gerais, Belo Horizonte, Brazil; 2 Embrapa Gado de Leite, Juiz de Fora, Brazil; 3 Center for Genetic Improvement of Livestock, University of Guelph, Guelph, Canada; 4 Beef Improvement Opportunities, Guelph, Canada; Universita degli Studi di Bologna, ITALY

## Abstract

Temperament is fundamental to animal production due to its direct influence on the animal-herdsman relationship. When compared to calm animals, the aggressive, anxious or fearful ones exhibit less weight gain, lower reproductive efficiency, decreased milk production and higher herd maintenance costs, all of which contribute to reduced profits. However, temperament is a trait that is complex and difficult to assess. Recently, a new quantitative system, REATEST^®^, for assessing reactivity, a phenotype of temperament, was developed. Herein, we describe the results of a Genome-wide association study for reactivity, assessed using REATEST^®^ with a sample of 754 females from five dual-purpose (milk and meat production) Guzerat (*Bos indicus*) herds. Genotyping was performed using a 50k SNP chip and a two-step mixed model approach (Grammar-Gamma) with a one-by-one marker regression was used to identify QTLs. QTLs for reactivity were identified on chromosomes BTA1, BTA5, BTA14, and BTA25. Five intronic and two intergenic markers were significantly associated with reactivity. *POU1F1*, *DRD3*, *VWA3A*, *ZBTB20*, *EPHA6*, *SNRPF* and *NTN4* were identified as candidate genes. Previous QTL reports for temperament traits, covering areas surrounding the SNPs/genes identified here, further corroborate these associations. The seven genes identified in the present study explain 20.5% of reactivity variance and give a better understanding of temperament biology.

## Introduction

Temperament is a complex trait comprising many phenotypes including curiosity, exploration, aggressiveness, reactivity, passivity, physical movements, persistent habits, emotions, alertness and response to novelty [1]. Like other behavioral traits, temperament is influenced by a complex network of interacting genetic components, environmental factors, genetic-environment interactions and life cycle events. In addition, the impact of parental experiences is transmitted not only through shared environment but also through epigenetic mechanisms [2]. Although temperament would seem to be a brain construction, it also reflects adrenal, thyroid, liver, muscle and peripheral nervous system metabolism [2–4].

Cattle temperament describes “consistent behavioral and physiological differences observed between individuals in response to human interaction or environmental challenge” [3,5]. An observable demonstration of cattle temperament is reactivity [6]. Reactivity to humans is mainly influenced by previous experiences of an animal with humans and depends on the context; for example, it can be influenced by fear [3]. Temperament is also an important component of bovine social behavior which affects animal welfare, animal-animal interactions and the animal-herdsman relationship. More excitable animals react with increased aggression and/or fear to human contact or handling. Aggressive animals present less weight gain, reduced reproductive efficiency, lower milk production, inferior meat quality and higher disease susceptibility [2]. They also cause more accidents, increase herd and facility maintenance costs; and, harm themselves, other animals and even herdsmen [7–9]. Consequently, animal temperament impacts the economic efficiency of the production system.

As expected, the broad and subjective definition of temperament leads to difficulties in the robust assessment of this trait [3,10]. Many different methods to evaluate different temperament phenotypes have been proposed [10]. In general, tests of temperament are divided into two categories: movement restraint or movement non-restraint methods. Non-restraint methods allow the animal to remain free to move around, while a technician subjectively assigns scores to the temperament or a device objectively measures traits that correlate to temperament, such as heart rate.

Among the restraint-based methods, the crush test and flight speed are frequently used. In the crush test, the animal is held in a crush and scores are subjectively assigned, considering the frequency and intensity of the movements, audible breathing and the frequency of bellowing and jumping [11]. In flight speed, the time the animal takes to travel a certain distance after being released from a balance-chute is measured using a photoelectric cell [12].

In the last decade, a new and objective method (REATEST^®^) was developed to evaluate animal reactivity [13]. In this test, the animal is held during the weighing and an electronic device, positioned under the chute, measures its reactivity based on the frequency and intensity of its movements while confined. This device contains an accelerometer which measures the frequence, intensity and temporary variation of movements for 20 seconds while the animal is on the chute. The total number of pulses is automatically processed on a specific software and converted in a value in a continuous grading scale. This value is used herein as the measurement of reactivity [13–15]. Flight speed and REATEST^®^ were compared in the evaluation of the Nellore breed temperament and presented high positive correlation [10]. In addition, REATEST^®^ results correlated better with temperament scores from crush test, than flight speed [13,15,16]. Similarly, Peixoto et al (2016) [14] found a significative correlation (0.89) between the crush test and REATEST^®^ in the Guzerat breed. REATEST^®^ has several advantages: 1) it does not interfere in the daily management of farms and does not increase the number of activities with the animal, thus allowing the assessment of many animals in one day; 2) it eliminates the subjectivity of the evaluator; 3) it allows the detection of higher phenotypic variability, when compared to temperament scores; and, 4) the test does not expose the evaluator to additional accident risks and requires no changes in farm structure [16].

REATEST^®^ has a main limitation, common to most phenotyping devices for behavior: lack of specificity. It has been stated that reactivity is related to aggressiveness in an animal [16]. Thinking about the reactions detected by REATEST^®^, it is reasonable that the device may also indirectly assess fear, panic, excitability, and anxiety. In this sense, reactivity, as a component of temperament has its own components.

Temperament is a complex trait, comprising many phenotypes, probably influenced by many genes and pathways as well as environmental factors and gene-environmental interactions [3]. Furthermore, within a population, the same phenotype (e.g., aggressiveness) may be caused by different genetic components among different individuals/breeds. In this context, the more complex the phenotype, the harder it is to find candidate genes/regions.

Despite its broad definition, several genes have already been associated with cattle temperament components [17–19], such as movement on a weighing scale, habituation, disposition and docility [17,18,20]. Genes as *GLRB* and *GRIA2* on BTA17 and *QKI* on BTA9 were already associated with flight speed [21] and *DRD4* (BTA29) with docility [22]. However, most of these studies have been carried out in taurine breeds [3,23].

In Brazil, most production systems are based on indicine breeds raised in pastures, a management system that implies less contact with humans. Due to this kind of management, but perhaps reflecting true biological differences, temperament issues have been reported for some indicine breeds [24]. Therefore, association studies aiming to discover the genetic basis of temperament variation in indicines would be helpful.

Guzerat is the third largest indicine purebreed in Brazil, exceeded in number only by Nellore and Gyr. In the first decades following its introduction in Brazil, Guzerat selection focused on meat but, over the last thirty years, breeders have been aiming at dual-purpose (meat and milk) selection. This breed has also been used frequently in crosses with both taurine and other indicine breeds to obtain animals with better performance for the tropical production systems.

Allegedly, Guzerat brings resistance to endo- and ectoparasites, endurance in adverse environments, particularly in the dry period or in the semiarid regions, satisfactory weight gain and growth, even consuming gross forage, and maternal ability [25]. However, temperament issues are occasionally reported, a risk exacerbated by the shape and size of the Guzerat horns. In this context, investigating the genetic basis of temperament in Guzerat is important, not only for scientific reasons, but also for practical ones. Herein we describe a genome-wide association study (GWAS) of reactivity as ascertained using the REATEST^®^ with a large sample of the Guzerat breed in Brazil.

## Materials and Methods

### Ethics Statement

This study was performed following approval of the Embrapa Dairy Cattle Ethical Committee of Animal Use (CEUA-EGL) under the protocol number 09/2014.

### Animals and Data

The data used in the present study is a part of the data collected and published by Peixoto et al (2016), where the reactivity of all females of five farms was evaluated [14]. The current sample was composed of 754 females, lactanting or not. Data were collected from five farms located in southeastern Brazil, distributed in areas having transitional vegetation from savannah-like (Cerrado) to rain forest (Mata Atlântica). These herds are part of a nationwide breeding program and represent an important genetic repository of the breed in Brazil. As usually seen in herd-based samples, the sample is not truly random and includes some related individuals. Indeed, as the selection program is partially based on a multiple ovulation-embryo transfer strategy, this sample includes, among other relationships, full- and half-sibs. Accordingly, proper methodologies were adopted in the analysis (see Genome-wide Association Study topic, below).

### Reactivity Evaluation

The reactivity of all females, from weaning to advanced ages (19–202 months) whether lactating or not, was measured, using REATEST^®^. This test uses an electronic device containing an accelerometer that is positioned under the chute to capture the cow’s movements for 20 seconds during the routine weighting. A computer program converts the frequency and intensity of movements to a value between 0 and 9,999 [13], herein referred to as REACT. Reactivity was measured twice in each herd, being one measure made in the rainy and the other made in the dry season of the year [14]. Here we report the results obtained in the dry season.

The Generalized Linear Mixed Model GLINMIX, a methodology available in PROC, Statistical Analysis System (SAS) v9.2 [26], was used to remove fixed effects from the reactivity data. The effects included were: herd, body weight (≤ 408 kg; ≥ 409 - ≤ 462 kg; ≥ 463 - ≤ 514 kg; > 514 kg), age (≤ 24 months; > 24 e ≤ 48 months; > 48 e ≤ 72 months; > 72 months), and physiological status (lactating or not). The effect of body weight was nested within age. Therefore, the model is described as:

$$y_{iklmn}=\mu +H_{i}+PS_{k}+A_{m}+EO_{l}+\varepsilon_{iklmn}$$ (1)

where, *Yiklmn* = dependable variable reactivity of the female *iklmn*, *Hi* = fixed effect of the herd *i*, *PSk* = fixed effect of the physiological status *k*, *EOl* = fixed effect of weight *l*, *Am* = fixed effect of age *m*; and *εiklmn* = residual random term in the observation *n*. This variable will be referred to as adjusted reactivity (REACT^adj^). The adjusted reactivity data was used in all the association tests performed in the following analyses (S1 Dataset).

### Genotyping

DNA was extracted from peripheral blood as described elsewhere [27]. Animals were genotyped using the Illumina BovineSNP50 v2 DNA Analysis BeadChip.

### Genotype Quality Control

The R open source software [28] was used to conduct statistical analysis. Genotype quality control was performed using the function *check*.*marker()* implemented in the package GenABEL [29]. Missing genotypes, sex errors and low quality markers were gradually excluded in an iterative process. Only markers with known position (54,060) in the bovine genome were checked. First, markers showing a minor allele frequency (MAF) less than 1%, call rate (CR) less than 95% and extreme deviations from Hardy-Weinberg Equilibrium (p-value<10^−6^) were excluded. Second, samples showing low mean call rates, high mean heterozygosity (FDR<1%) and high identity by state (IBS>95%) across a random sample of markers were excluded. This procedure was repeated until there were no more inconsistencies in the sample. Details on markers and animals excluded in each step are provided on Tables A and B in S1 File. Due to the low coverage of the sexual chromosomes in the SNP chip, only autosome markers were used. Therefore, 31,387 polymorphic markers, positioned according to the UMD_3.1 bovine assembly map, and 754 individuals were used for GWAS.

### Genome-wide Association Study

In the GWAS, the GRAMMAR-Gamma method implemented in the GenABEL package was used [30]. GRAMMAR-Gamma is a two-step mixed model, which assumes that each SNP has a small effect on the phenotype and, thus, on heritability (*h*^2^). Therefore, it calculates the estimates of *h*^2^ in the first step and applies the resulting matrix to the association test. Additionally, the association results are corrected for genomic control and for deviations in the estimates of effects. Markers are fitted as a linear covariate in the statistical model and the genotypes are represented as the number of copies of the less frequent allele. The genomic relationship matrix used to obtain the heritability estimates was calculated through the *ibs()* function implemented in the GenABEL package. The GRAMMAR-Gamma test accounts for population stratification and familial relationship in the sample. No additional fixed effects were included in the second step of the analysis, as the adjusted reactivity (REACT^adj^) were used in the score test. Values of inflation factor (λ) between 0.9 and 1.1 were considered acceptable and the test statistics were divided by λ to adjust for the remaining inflation [30].

### Correction for Multiple Testing

A false discovery rate (FDR) threshold set to 5% was used to correct the results obtained in the GWAS for multiple testing.

### Gene Mapping and In-silico Functional Analyses

Map positions of the SNPs were based on UMD3.1. Information about the markers (rs, map position, alleles) were obtained using the SNPchiMp tool [31]. When these SNPs were located within a gene, several analyses were carried out. First, the position of the SNP (intronic or exonic, 5´-UTR or 3´-UTR) was determined. Second, repercussions of the allele substitution on splicing and miRNA recognition sites were predicted. Third, a list of the genes contained within a 250 kb interval, upstream and downstream of the marker, was obtained using the NCBI (http://www.ncbi.nlm.nih.gov/) and Ensembl (http://www.ensembl.org/index.html) databases. When the markers map to intergenic regions, analyses of evolutionary conservation of a SNP nucleotide position and a search for evolutionary conserved regions (ECR) around them were performed using the software Multi Pip Maker [32] and the ECR Browser in USCS [33], respectively. A conserved region was considered as an ECR core, when it presented more than 350bp length and more than 77% of similarity with at least four of the eight species compared with *Bos taurus* herein (fugu, tetraodon, frog, chicken, opossum, mouse and human). These species were selected based on the availability of their pre-computed alignment in the ECR Browser software (all available species until April 2015 were selected). When an ECR was encountered, a search for transcription factor binding sites (TFBS) inside the ECR was performed using rVista 2.0 [34] in order to predict possible regulatory sites nearby the SNP associated with REACT^adj^. Information on each gene of the list was obtained from different sources, such as articles published in PUBMED (http://www.ncbi.nlm.nih.gov/pubmed/), UNIPROT (http://www.uniprot.org/), GeneCards (http://www.genecards.org/), and STRING (http://string-db.org/).

## Results

REACT presented a skewed distribution distribution, with most animals presenting low reactivity scores; only 189 individuals, 25% of sample, showed REACT>1010 or REACT^adj^>0.2857. Therefore, the Generalized Linear Mixed Model GLINMIX was used to obtain a skew-normal distribution for data analysis. Mean, minimum, maximum and the heritability of both REACT and REACT^adj^ are presented in Table 1.

**Table 1:** Heritability and descriptive statistics for REACT and REACT^adj^.

Trait	Animals	Median	SD	Minimum	Maximum	Heritability
**REACT**	754	470	814	161	7,945	0.2899
**REACT^adj^**	754	-0.167	0.725	-0.888	5.213	0.1388

Note—SD—standard deviation; REACT is the score obtained in the REATEST^®^ and REACT^adj^ is a variable obtained through the Eq (1), above.

Mean values of marker distribution ranged from 10 to 14 markers/Mb for all the autosomes. Mean heterozygosity by sample was 0.26. GRAMMAR-Gamma was used, returning an inflation factor for the test (λ = 1.047) within the usually accepted limits of 0.9 and 1.1 (Fig A in S1 File). In addition, no significant LD between non-adjacent markers was detected.

**Fig 1:**
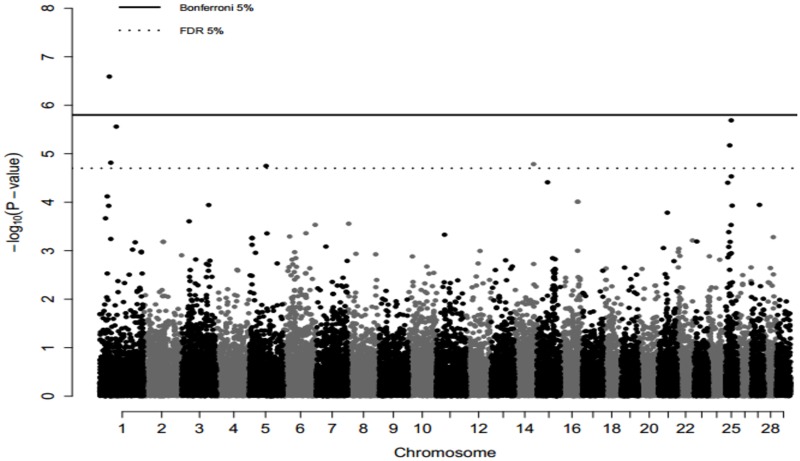
Manhattan plot of the -log10(p-values) for genome-wide association of REACT^adj^. The continuous line represents the 5% Bonferroni threshold (1.59 x 10^−6^) and the dashed line represents the 5% FDR threshold.

After an FDR correction (α = 5%), seven markers were associated with REACT^adj^, mapping to chromosomes BTA1, BTA5, BTA14 and BTA25 (Table 2). One of these markers (rs109007595, p = 2.56 x 10^−7^), was significantly associated with REACT^adj^ even after a Bonferroni correction (α = 5%) (Fig 1). rs109007595 maps on BTA1 at the 35,014,129 bp position and presented an estimated allele substitution effect of 0.859 units in REACT^adj^. According to UMD_3.1, five of the seven SNPs described above map to introns (one of them maps simultaneously to an intron and to the upstream region of the gene, depending on the isoform). No significant repercussion in splicing or miRNA recognition sites were predicted as a consequence of these allele substitutions. The other two SNPs map to intergenic regions. rs29002595 maps 141 kb upstream to netrin 4 (*NTN4*) and rs41965198 maps to over 250 kb upstream to ephrin receptor A6 (*EPHA6*). For these SNPs, analyses of evolutionary conservation were conducted. No significant results were for rs29002595. rs41965198 affects a nucleotide position which is conserved in humans, dogs, pigs and chimpanzees, but not in chickens and mice.

**Table 2:** Significantly associated SNPs for REACT^adj^ in Guzerat obtained by the GRAMMAR-Gamma method.

SNP reference	BTA	Position (bp)	Alleles	Genes	Region	MAF^1^	p-value	Multiple testing correction	PVE^2^	PHE^3^	Allele Substitution Effect^4^
**rs109007595**	1	35014129	T,C	*POU1F1*	Intronic	0.016	2.56e-07	Bonferroni5%	0.037	0.269	0.856
**rs41965198**	1	40353369	C,T	*LOC782966*, *EPHA6*	Intergenic	0.012	1.58e-05	FDR5%	0.026	0.188	0.823
**rs108944043**	1	60231667	A,G	*ZBTB20*	Intronic	0.017	1.53e-06	FDR5%	0.031	0.223	0.763
**rs42063418**	25	14541927	A,G	*ABCC1*	Intronic	0.047	6.73e-06	FDR5%	0.028	0.202	0.441
**rs109589165**	25	19995956	C,T	*VWA3A*	Intronic	0.013	2.05e-06	FDR5%	0.031	0.226	0.877
**rs110729726**	14	72106554	C,T	*KIAA1429*	Intronic	0.068	1.67e-05	FDR5%	0.026	0.188	0.355
**rs29002595**	5	60513092	C,T	*NTN4*	Intergenic	0.011	1.8e-05	FDR5%	0.026	0.186	0.866

^1^ Minor allele frequence for each locus.

^2^ Portion of the variance that is explained by the SNP.

^3^ Portion of the heritability that is explained by the SNP.

^4^ Allele substitution effect in REACT^adj^ measured in points.

In the search for ECRs, eight species were used in the multi-alignment: fugu, tetraodon, frog, chicken, opossum, mouse, chimpanzee and human. An 800kb fragment upstream from the closest gene (*EPHA6*), which included the SNP, was aligned in all these species. Three ECRs were identified around the rs41965198 position. ECR1 (chr1:40280223–40281010) is conserved in mammals and in chicken. ECR2 (chr1:40221973–40222560) is conserved in chicken and in mammals, except for opossum. ECR3 (chr1:40018913–40019884) is conserved in mammals, chicken and frogs. Analyses of TFBS in the three deeply conserved ECRs returned a large number of predicted sites inside the ECRs, indicating that they could be involved in regulatory processes.

## Discussion

Over the last decade, GWAS helped to identify underlying genes for many complex characteristics. Mapping candidate regions for a complex trait such as temperament or reactivity requires considerably large samples in order to detect significant associations with the many different underlying genetic variants. Alternatively, mapping efforts can be conducted using specific subpopulations in which the trait and its genetic etiologic factors are less complex. This strategy has been successful for deconstructing and mapping complex behavioral traits in humans [35]. The disadvantage of this approach is that a candidate genetic variation, identified in a subpopulation, may not be a major etiologic component in the breed, the species, or in general.

In this context, the Guzerat provides an interesting model for studying such characteristics because its population is relatively small and was founded by a limited number of ancestors imported to Brazil since the end of the nineteenth century. Guzerat has also passed through a series of bottleneck events, due to its use in crossbreeding [36]. Therefore, Guzerat has a more restricted genetic background and, consequently, a probably less complex genetic component for reactivity.

Another relevant aspect in a genetic association study is the heritability of a trait. Heritability estimates of behavioral traits vary widely depending on the experimental design [37]. Temperament heritability estimates range from 0.08 to 0.53 in experiments using reactivity tests with a mobile scale [16,37], flight speed [38,39], crush score [40] and flight distance [41]. In some Guzerat herds, heritability estimates may also reflect the influence of taming [42] and selection against the more aggressive animals.

Several association studies for cattle temperament have already been published and reviewed by Haskell and colaborators in 2014 [23]. QTLs on BTA1 were identified in several studies mapping temperament phenotypes, such as movement on a weighting scale [17], habituation in beef cattle [17], disposition in Brahman x Angus crossbreeds [18], and docility in German Simmental and German Angus breeds [20]. QTLs on BTA16, BTA8, BTA4 and BTA29 have been repeatedly associated with temperament. In addition, QTLs for temperament phenotypes have occasionally been reported on BTA2, BTA3, BTA5, BTA9, BTA11, BTA12, BTA14, BTA15, and BTA18. Nevertheless, only a small number of genes has already been suggested as candidates. For instance, *DRD4* (BTA29) has been associated with docility. *GLRB* and *GRIA2* (both in BTA17) and *QKI* (BTA9) have been associated with flight speed [21,22]. Three of the seven QTLs detected in the present study map up to 5Mb to previously reported QTLs [23,43]. In BTA1, rs109007595 maps close to a QTL for temperament; in BTA25, rs42063418 maps close to a QTL for aggressiveness and rs109589165 maps close to a QTL for social separation vocalization. [23]. Below, the QTLs identified in the present study will be discussed in detail.

### BTA1

In BTA1, significant results were obtained for three markers distributed over a 25Mb interval (Table 2). It is a gene-rich interval, containing approximately 110 genes. rs109007595 (Table 2; Fig 1), the marker significantly associated even after a Bonferroni correction, maps within intron 2 of POU1F1 (Pituitary-specific positive transcription factor 1), a gene encoding a member of the POU family of transcription factors that regulate mammalian development. Its protein, PIT1, regulates the expression of several genes involved in pituitary development and expression of prolactin and TSHβ. Therefore, POU1F1 is essential for nervous system development, body growth and hormone balance [44]. In the present study, this marker explains 3.7% of the variance, and the allele substitution effect shows an average increase of 0.856 points in REACTadj. This makes POU1F1 an important candidate for this trait.

In humans, *POU1F1* mutations cause anterior hypopituitarism (deficiency of prolactin, thyrotropin and the growth hormone) [45,46] and absence/delay of adrenarche and pubarche [47]. *POU1F1* has already been identified as QTL for production traits in bovines. In cattle, *POU1F1* polymorphisms have been associated with a wide range of production traits such as: milk production; birth weight; weight at 90, 270 and 450 days; weaning weight; and, pre- and post-weaning average daily weight gains [48,49].

On the other hand, some studies have already described an association between the selection for high production efficiency in farm animals and undesirable side effects such as loss of homeostatic balance, resulting in pathologies and affecting animal welfare [50,51]. Despite the positive effects of *POU1F1* mutations on production traits, there are, nevertheless, negative impacts on reproduction phenotypes. These negative effects may implicate *POU1F1* as a candidate for the negative hitchhiking effects observed in milk selection programs. However, an association with reactivity has not yet been described.

The second significantly associated marker, rs41965198, maps to an intergenic region 5Mb downstream from *POU1F1*. This marker explains 2.6% of the reactivity variance, with an allele substitution effect of 0.823 points in REACT^adj^. The genes closest to this marker are *LOC782966* (*tubulin alpha-1A chain*) and *EPHA6*, mapping over 200 kb downstream from rs41965198. *LOC782966* (*tubulin alpha-1A chain*) is a retrogene and there is no information in the literature or in databases confirming that it is expressed.

*EPHA6* encodes for Ephrin type-A receptor 6. In mouse embryos, this receptor is highly expressed during the development of the central nervous system; and, in adult animals, in the hypothalamus, thalamus and amygdala. EPHA6 is a tyrosine kinase receptor which binds promiscuously the GPI-anchored ephrin-A family ligands to adjacent cell membranes, leading to contact-dependent bidirectional signaling between neighboring cells. The signaling pathway activated by the ephrin-A-EPHA6 ligation mediates processes such as axon guidance and axon growth repulsion [52], indispensable components of central nervous system development. EPHA6 function has been described extensively in eye development, where mutations were related to mouse retina malformations [53]. In addition, *EphA6* KO mice present abnormally low results in tests evaluating learning and retrieval of the fear conditioning stimulus [54].

The existence of three deeply conserved ECRs suggests that the region around rs41965198 may contain long distance regulatory elements for the nearby genes. Alternatively, rs41965198 may be in linkage disequilibrium with the truly functional variant, since this polymorphism does not map to these ECRs. To test this hypothesis, an analysis of TFBS was performed on these three ECRs and the results suggest that this SNP does, in fact, map close to a probable long-range regulatory element [55,56].

rs108944043 is located within the first intron of the gene *ZBTB20* (*zinc finger and BTB domain containing 20)* 25Mb downstream from *POU1F1*. This gene encodes for a transcription factor that has been implicated in hematopoiesis, oncogenesis, and immune response in mammals. Diseases associated with *ZBTB20* in humans include Primrose syndrome and juvenile pilocytic astrocytoma. This gene is more abundantly expressed in tissues from the nervous, secretory and reproductive systems in humans (http://www.uniprot.org/uniprot/Q9HC78). In the brain of transgenic mice, ectopic *Zbtb20* expression produces cortex lamination defects resulting in behavioral abnormalities, suggesting impaired processing of visual and spatial memory [57]. *Zbtb20* directly impacts on the development of different parts of the hippocampus [58], affecting behavioral traits such as memory and anxiety [59]. This marker explains 3.1% of the trait variance; and, the allele substitution effect is an increase of 0.877 points in REACT^adj^. Therefore, *ZBTB20* is a new, highly pleiotropic and interesting candidate for reactivity.

On the other hand, analyzing the *ZBTB20* neighborhood, another important functional candidate gene emerges, *dopamine receptor 3* (*DRD3*), 70kb upstream from *ZBTB20*. *DRD3* is highly expressed in the ventral striatum, a region associated with behavioral traits, and poorly expressed in other regions of the central nervous system [60]. *DRD3* has been associated with high impulsiveness in violent individuals and also with sensory sensitivity (the capacity to react to sensory stimuli with low stimulating value). Furthermore, DRD3 blockers induce cognition-enhancing and hyperactivity-dampening effects [61–63].

### BTA25

On BTA25, significant results were obtained for two markers mapping 5Mb from each other (Table 2). rs42063418, associated with REACT^adj^ at FDR 5%, is located within intron 18 of *ABCC1*, a membrane-associated protein, member of the ATP-binding cassette (ABC) superfamily, highly expressed in the brain and involved in multidrug resistance. ABCC1 is a component of the blood-brain barrier. In addition, it exports corticosteroids, which have been involved in stress response, from the adipose tissue [64]. Therefore, it may influence temperament through at least two different mechanisms. The marker within *ABCC1* explains 2.8% of the REACT^adj^ variance, with an allele substitution effect of 0.441 points.

The second marker in this region, rs109589165, showed the second lowest p-value in this GWAS (p-value = 2.05E-06, significant at a 5% FDR threshold). This SNP is located within the *von Willebrand factor A domain containing 3A* (*vWA3A*) gene. Depending on the transcript isoform, it is located in the intron 1, 2, or even before the 5’-UTR. Proteins containing von Willebrand domains are involved in basal membrane formation, cell migration, cell differentiation, adhesion, haemostasis, signaling, chromosomal stability, malignant transformation and immune defenses. Although vWA3A is differentially expressed in the blood, brain, lungs, ovaries and testes [65], there is still no evidence about any function of this gene in behavior. The marker within *ABCC1* explains 3.1% of the REACT^adj^ variance, with an allele substitution effect of 0.877 points.

### BTA14

rs110729726 is located inside *KIAA1429* intron 1. This gene codes for a spliceosome-associated protein that is putatively involved in mRNA methylation and splicing regulation [66]. This is the first evidence of *KIAA1429* involvement in temperament. This SNP explains 2.6% of REACT^adj^ variance and has an allele substitution effect of 0.355 points.

### BTA5

rs29002595 is the last marker associated with REACT^adj^ over the 5% FDR threshold. This marker maps to a gene-rich region on BTA5. Two functional candidate genes were identified in this region. This marker maps 10kb upstream from the *Netrin 4 (NTN4)* gene, which codes for a member of the family of laminin-related proteins. Netrins are involved in axon guidance and cell migration during development [67]. NTN4 or beta-netrin increases both the length and number of neurites in rat olfactory bulb cultures [68]. Therefore, *NTN4* acts directly on axon development and morphogenesis, making it an interesting candidate for reactivity.

Another gene in this region, *SNRPF*, also codes for a spliceosomal protein. Alterations in the RNA binding function have already been associated with behavioral disturbances in mice [69]. In humans, *SNRPN* deletions cause Prader-Willi syndrome, characterized by polyphagia and temper tantrums, which are time-limited crises of aggressive and violent behavior that subside, succeeded by the calm and agreeable temperament described for these patients as typical [70]. rs29002595 explains 2.6% of REACT^adj^ variance; the allele substitution effect is 0.866 points.

As most of the associated markers in the present study are intronic, we tested for the presence of miRNA recognition sites and alternative splicing using the programs miRBase and ASSP (Alternative Splice Site Predictor). No significant allele substitution effects produced by these SNPs were identified. Therefore, the QTLs identified here probably reflect the effects of variants, in linkage disequilibrium to the SNPs tested, which contribute to reactivity in Guzerat [71–74].

## Conclusion

This is the first study to use GWAS to investigate the genetic basis of reactivity in Guzerat. The QTLs identified here are located inside or close to genes such as *POU1F1*, *DRD3*, *VWA3A*, *ZBTB20*, *EPHA6*, *SNRPF* and *NTN4* implicated in the development or function of the neural system. They are, therefore, strong candidates for temperament phenotypes, more specifically, reactivity, anxiety and aggression. Together, these QTLs explain approximately 20.5% of reactivity variance, give a better understanding of temperament biology and open possibilities for new studies in the field.

## Supporting Information

S1 File **Fig A**—Quantile—quantile plot of observed and expected—log10(p-values) obtained in GRAMMAR-Gamma association analysis. **Box A**—Exclusion criteria for markers and samples. **Table A**—Markers excluded from analysis. **Table B**—Individuals excluded from analysis.
(DOCX)

S1 Dataset **File REACTadj_110117.txt**–REACT^adj^ values per individual. **File GWASresults_110117.txt**–GWAS results for each marker tested in the Grammar-gamma model.
(ZIP)
