# Supplementary material for: Identification of Candidate Genes for Reactivity in Guzerat (Bos indicus) Cattle: A Genome-Wide Association Study
Source: PLoS One. 2017 Jan 26;12(1):e0169163. doi: 10.1371/journal.pone.0169163 (PMC5268462; doi:10.1371/journal.pone.0169163)
Supplement: S1 File — Fig A—Quantile—quantile plot of observed and expected—log10(p-values) obtained in GRAMMAR-Gamma association analysis. Box A—Exclusion criteria for markers and samples. Table A—Markers excluded from analysis. Table B—Individuals excluded from analysis. (DOCX) [file pone.0169163.s001.docx]

# S1 File

Figure A


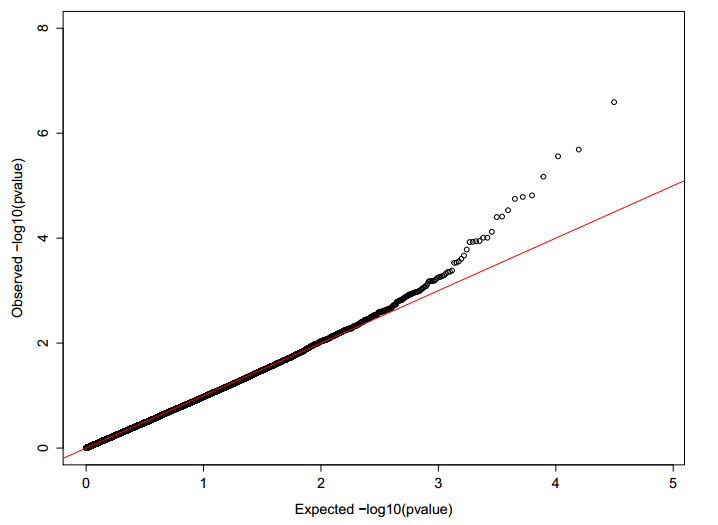


**Figure A -** Quantile–quantile plot of observed and expected –log10(p-values) obtained in GRAMMAR-Gamma association analysis.

**Box A –**Exclusion criteria for markers and samples

Using check.marker() procedure, 53 markers (CR<0.1) and 774 markers (0.1<CR<0.95) were excluded due to low call rate; 637 markers were excluded due to low call rate associated with low MAF (CR<0.95 and MAF<0.01); 1,168 markers were excluded because they did not fit HWE expectations (P<1e-06), associated or not with low call rate and/or low MAF; and, 19,420 markers were excluded only due to low MAF (MAF<0.01). Therefore, 31,999 markers were used in the analyses (Supplementary table 1).

In addition, six individuals were excluded due to low mean call rate per sample (CR<0.95); of these, three also presented high autosomal heterozygosity (FDR<1%). One individual was excluded only due to high autosomal heterozygosity (FDR<1%) and three other individuals were excluded due to high IBS compared to other individuals from the sample (IBS>0.95) (Supplementary table 2).

Table A - Markers excluded from analysis

|  | Low CR (CR<95%) | Low MAF (MAF<0.01) | Out of HWE expectations (P<1e-06) |
| --- | --- | --- | --- |
| Low CR (CR<95%) | 827 | 637 | 825 |
| Low MAF (MAF<0.01) | NA | 19420 | 14 |
| Out of HWE expectations (P<1e-06) | NA | NA | 329 |

Note - CR: Call Rate; MAF: Minor Allele Frequency; HWE: Hardy-Weinberg Equilibrium; NA: Not Applicable.

Table B - Individuals excluded from analysis

|  | Low CR (CR<95%) | Out of Heterozygosity expectations (FDR<1%) | High IBS (>0.95) |
| --- | --- | --- | --- |
| Low CR (CR<95%) | 3 | 3 | 0 |
| Out of Heterozygosity expectations (FDR<1%) | NA | 1 | 0 |
| High IBS (>0.95) | NA | NA | 3 |

Note - CR: Call rate; FDR: False Discovery Rate; IBS: Identity By Descendent; NA: Not Applicable
